# Direct costs of hypertensive patients admitted to hospital in Vietnam– a bottom-up micro-costing analysis

**DOI:** 10.1186/s12913-014-0514-4

**Published:** 2014-10-28

**Authors:** Thi-Phuong-Lan Nguyen, Thi Bach Yen Nguyen, Thanh Trung Nguyen, Van Vinh Hac, Hoa H Le, CCM Schuiling-Veninga, Maarten J Postma

**Affiliations:** Department of Pharmacy, Unit of PharmacoEpidemiology & PharmacoEconomics (PE2), University of Groningen, Antonius Deusinglaan 1, building 3214, room 0461, Groningen, the Netherlands; Department of Health economic, Ha Noi University of Medicine, Ha Noi, Vietnam; Thai Nguyen University of Medicine and Pharmacy and Thai Nguyen general hospital, Thai Nguyen, Vietnam; Faculty of Public Health, Thai Nguyen University of Medicine and Pharmacy, Thai Nguyen, Vietnam; Department of Epidemiology, University Medical Center Groningen, Groningen, the Netherlands

**Keywords:** Cost analysis, Hypertension, Screening, Vietnam

## Abstract

**Background:**

There is an economic burden associated with hypertension both worldwide and in Vietnam. In Vietnam, patients with uncontrolled high blood pressure are hospitalized for further diagnosis and initiation of treatment. Because there is no evidence on costs of inpatient care for hypertensive patients available yet to inform policy makers, health insurance and hospitals, this study aims to quantify direct costs of inpatient care for these patients in Vietnam.

**Methods:**

A retrospective study was conducted in a hospital in Vietnam. Direct costs were analyzed from the health-care provider’s perspective. Hospital-based costing was performed using both bottom-up and micro-costing methods. Patients with sole essential or primary hypertension (ICD-code I10) and those comorbid with sphingolipid metabolism or other lipid storage disorders (ICD-code E75) were selected. Costs were quantified based on financial and other records of the hospital. Total cost per patient resulted from an aggregation of laboratory test costs, drug costs, inpatient-days’ costs and other remaining costs, including appropriate allocation of overheads. Both mean and medians, as well as interquartile ranges (IQRs) were calculated. In addition to a base-case analysis, specific scenarios were analyzed.

**Results:**

230 patients were included in the study (147 cases with I10 code only and 83 cases with I10 combined with E75). Median length of hospital stay was 6 days. Median total direct costs per patient were US$65 (IQR: 37 -95). Total costs per patient were higher in the combined hypertensive and lipid population than in the sole hypertensive population at US$78 and US$53, respectively. In all scenarios, hospital inpatient days’ costs were identified as the major cost driver in the total costs.

**Conclusions:**

Costs of hospitalization of hypertensive patients is relatively high compared to annual medication treatment at a community health station for hypertension as well as to the total health expenditure per capita in Vietnam. Given that untreated/undetected hypertension likely leads to more expensive treatments of complications, these findings may justify investments by the Vietnamese health-care sector to control high blood pressure in order to save downstream health care budgets.

**Electronic supplementary material:**

The online version of this article (doi:10.1186/s12913-014-0514-4) contains supplementary material, which is available to authorized users.

## Background

Hypertension represents a health and economic burden worldwide. In 2000, approximately one quarter of the adult populations had hypertension, equaling to approximately 972 million adults [1]. Furthermore, it has been projected that almost 30% of the world’s adult population will be hypertensive by 2025 [1]. High blood pressure and associated diseases may be responsible for up to 7 million deaths annually worldwide [2]. In the Eastern European and Central Asian regions, high blood pressure is estimated to directly or indirectly account for 25% of all health expenditures [3]. In Southwest China, a cost-of-illness analysis from the societal perspective in 2010 estimated the cost of hypertension to be US$9,393 per patient [4]. In the Philippines, a health insurance company reported reimbursement for hypertension- related diagnoses during 3.5 years to be US$56 million for 360,016 patients [5]. This equals to 34% of their financial budget for hospital spending [5].

Among the group of developing countries, the prevalence of hypertension in Vietnam can be rated as intermediate with estimates ranging from 20% to 30% in adults [6]. In recent years, hypertension has been one of the main contributors to the overall burden of disease in Vietnam [7,8]. A national survey, conducted between 2002 and 2008, estimated prevalence rates for overall, male, and female populations to be 25.1%, 28.3% and 23.1%, respectively [8]. Using the Framingham general cardiovascular risk score, it was estimated that the prevalence of people with overall 10-year cardiovascular disease (CVD) risks ≥10%, 20% and 30% are 27%, 10% and 3.9%, respectively [9,10] For patients who are 60-and-above years old, hypertension as the underlying cause of death ranks third for males (6.2% of all deaths) and second for females (6.6% of all deaths). For all ages, hypertension ranks sixth for males (3.8% of all deaths) and second for females (5.1% of all deaths) [7]. Treatment of hypertension is crucial to avert these high risks, associated costs and related deaths.

Compared to surrounding countries, hypertension may impact even higher costs to society in Vietnam because diagnosis and initiation of treatments for hypertension often take place in the hospital. This may be explained by several factors, including the absence of some specific services in primary care for all regions of the country, the perception that hospital care offers a higher quality of care and the preference for specific services to be in a hospital setting for administrative and convenience reasons. At present, the inpatient and outpatient economic burdens of hypertension in Vietnam have not been estimated precisely. In addition, studies on the topic are scarce. One study at the community-health station level on the cost of drug treatment for the whole population over a 10-years period estimated costs of 9,808 billion VND associated with grade 1 hypertension and 11,192 billion VND associated with grade 2 and 3 hypertension [11]. The sum of these figures represent approximately 14% of the total health expenditure in 2010 [12,13]. Notably, among hypertensive patients, only 29.6% was treated and 10.7% achieved target blood pressure control [8]. Patients, who are not treated and have uncontrolled high blood pressure, are at higher risks of complications requiring hospitalization, which add to the economic burdens for the health care system.

The health-care provider payment system in Vietnam is currently being reformed. The fee- for-service system will be replaced by case-mix payments and eventually diagnostic-related groups [14]. Thus, to support reimbursement decisions based on diagnostic-related groups, it would be useful for the Vietnamese health insurance system to have accurate estimates of costs associated with hypertension.

In addition, hospitals in Vietnam are gaining greater autonomy. Having knowledge on their expenditures for inpatient care services will help these hospitals to improve their financial management as well as to adequately issue fees for both insured and non-insured patients. Currently, neither information on the costs of inpatient care for the treatment of hypertension and its consequences nor solid estimates on the comprehensive costs of hypertension management is available in Vietnam.

To address these gaps of information, we conducted this study to quantify the direct costs of inpatient care for hypertensive patients. Furthermore, these results may 1) contribute to a better understanding of the economic burden of hypertension in Vietnam, 2) help with reimbursement decision making for insured patients, 3) support setting potential fees and charges to be issued for non-insured patients and 4) inform on potential impacts of preventive policies.

## Methods

### Study design and setting

We conducted a retrospective study in the Thai Nguyen hospital. Data was collected from the financial records during October 1st 2010 to September 30th 2011.

The study was conducted at a regional hospital with 800 beds in the city of Thai Nguyen, which is located in a mountainous area that is approximately 100 km North of Hanoi. It serves patients from Thai Nguyen and neighboring provinces.

All costing of resource utilization was adjusted to 2011 levels and presented in US$ using the exchange rate of US$1 to VND20.830 [15].

### Study participants

Using the International Classification of Diseases 10^th^ version (ICD-10), we identified and retrieved information on all patients with codes I10 alone (essential or primary hypertension) or comorbid patients with I10 combined with E75 (sphingolipid metabolism and other lipid storage disorders). The latter group was included because hypertension often coincides with these disorders. The combination of diagnoses might reflect a relatively large share of the overall hypertensive patient group with deviating costs. ICD-10 codes were taken from hospital databases and individual patients’ records.

Information on age, sex and ICD code was available for all of the selected patients with ICD code I10 or I10 combined with E75.

However, data might be missing as administrators may forget to enter classification data or misclassify; i.e. patients and/or patient-related data might be missing. In addition, by doing this retrospectively, we had no certainty on whether each financial record was included or not. Nurses should enter all consumed items such as drugs, tests, medical materials into the database system before she/he can get them from store or lab services. Other cost items such as patient-days and examinations were automatically recorded for every patient. This ensures that all these items were recorded and financial records are likely complete. Whenever financial records seemed grossly incomplete or absent at all, patients were excluded. This was however not the case. It was therefore plausibly assumed that missing data was limited and random. Thus, no specific bias was expected.

### Costing perspective and cost components

Direct costs were analyzed from the provider perspective. Data on costs included all charges to patients for drugs, materials (both medical and non-medical) and laboratory testing, all crucial elements in the financial records and reflecting adequate charging. In particular, hospital-based costing was performed using both bottom-up and micro-costing methods and aggregation was subsequently conducted for all costs related to medical services used by a group of hospitalized patients [16,17]. For this purpose, costs of inpatient care were broken down into two parts:

Inpatient – days costs and other costs (laboratory testing, drugs, medical materials charged to patients directly, admission, and examinations by specialists). Costs of inpatient care can be expressed as:

$$ \begin{array}{c}\mathrm{Costs}\ \mathrm{o}\mathrm{f}\ \mathrm{inpatient}\ \mathrm{care}=\mathrm{inpatient}\hbox{-} \mathrm{days}\ \mathrm{cost}\mathrm{s}+\mathrm{laboratory}\ \mathrm{t}\mathrm{est}\ \mathrm{cost}\mathrm{s}+\mathrm{drug}\ \mathrm{cost}\mathrm{s}+\\ {}\kern0.1em \mathrm{cost}\mathrm{s}\ \mathrm{o}\mathrm{f}\ \mathrm{medical}\ \mathrm{materials}\ \mathrm{charged}\ \mathrm{t}\mathrm{o}\ \mathrm{patients}\ \mathrm{directly}+\mathrm{admission}\ \\ {}\mathrm{cost}+\mathrm{costs}\ \mathrm{o}\mathrm{f}\ \mathrm{examinations}\ \mathrm{b}\mathrm{y}\ \mathrm{s}\mathrm{pecialists}\end{array} $$

### Method of cost measurements

#### Calculation of inpatient – days’ costs

The step-down allocation method- partially adjusted for interaction between overhead departments- was applied for allocating overhead costs [17]. In the base-case, the discount rate for medical equipment and building was 3% [17]. The formula applied here may be expressed as:

$$ \begin{array}{l}\mathrm{Total}\ \mathrm{costs}\ \mathrm{o}\mathrm{f}\ \mathrm{each}\ \mathrm{department} = \mathrm{t}\mathrm{o}\mathrm{t}\mathrm{al}\ \mathrm{labor}\ \mathrm{costs}\ \\ {} + \mathrm{t}\mathrm{o}\mathrm{t}\mathrm{al}\ \mathrm{costs}\ \mathrm{o}\mathrm{f}\ \mathrm{materials}/\mathrm{infrastructure}\ \left(\mathrm{both}\ \mathrm{medical}\ \mathrm{and}\ \mathrm{n}\mathrm{o}\mathrm{n}\hbox{-} \mathrm{medical},\ \mathrm{n}\mathrm{o}\mathrm{t}\ \mathrm{charged}\ \mathrm{t}\mathrm{o}\ \mathrm{patients}\ \mathrm{directly}\right)\ \\ {} + \mathrm{t}\mathrm{o}\mathrm{t}\mathrm{al}\ \mathrm{costs}\ \mathrm{o}\mathrm{f}\ \mathrm{capital}\ \left(\mathrm{both}\ \mathrm{medical}\ \mathrm{and}\ \mathrm{n}\mathrm{o}\mathrm{n}\hbox{-} \mathrm{medical}\ \mathrm{equipment}\ \mathrm{and}\ \mathrm{buildings}\right)\end{array} $$

Labor costs were calculated based on the actual payment for labor by the hospital every month during a year; funding was provided by government or specific funds for services organized by the hospital. Material/infrastructure costs were calculated by items used at each department multiplied with the price market which the hospital paid based on again monthly updated records of the hospital. Capital costs were calculated based on assets’ records of the hospital, which is updated every year.

Notably, non-medical costs of materials/infrastructure primarily comprise of power, telephone, uniforms, stationary and cloths. In the absence of any detailed information, an assumption was made that equal costs for an inpatient day would apply for every disease within each department.

The inpatient–day costs per department resulted from the total costs divided by the total number of inpatient days for that department:

$$ \mathrm{Inpatient}\ \hbox{--}\ \mathrm{day}\ \mathrm{costs} = \frac{\mathrm{Total}\ \mathrm{costs}\ \mathrm{a}\ \mathrm{given}\ \mathrm{department}}{\mathrm{total}\ \mathrm{number}\ \mathrm{of}\ \mathrm{patient}\hbox{-} \mathrm{days}\ \mathrm{a}\mathrm{t}\ \mathrm{t}\mathrm{hat}\ \mathrm{department}} $$

### Other costs and total patient costs

The cost-to-charge ratio method was applied to calculate laboratory testing costs [17]. While the input data to calculate cost of each test - such as number of chemicals used, time investment of staff members to run each test, etcetera - were limited, total input of each department, total number of tests per type and prices were available. Therefore, cost-to-charge ratio was considered to be the best method for calculating laboratory costs. At each laboratory department, we tracked the number of each test and multiplied these by the charges that apply in the hospital (base on the recommendation of the Ministry of Health). We subsequently summed up all those items to identify total finances of that department. Cost-to-charge ratio in each department was calculated dividing total input costs of each department in terms of building, labor, material, equipment, etcetera by those total finances generated through charges. Information on numbers of test was only available for the last half of the year because of a mistake in the software to extract total number of each test. To annualize, the total numbers for each test was multiplied by two.

In this study, standard national prices for drugs and materials were not available. As Thai Nguyen hospital is a non-profit hospital, we assumed that the use of drug and material charges from this hospital would well approach actual costs. Cost of drugs and materials were calculated based on the current market prices, which the hospital paid. Therefore, we could validly impute these charges into our cost analysis for each drug and material used [17]. Both drugs and medical materials were charged to patients directly and accordingly inserted in the analysis. These costs were consistently calculated based on numbers of items used multiplied by the specific prices of each item.

Admission cost, which is required for every intake examination for admission to the hospital, was calculated similarly as was done for inpatient – day’ costs. Admission costs per patient is equal to the total costs of outpatient department divided by the total number patient visits at outpatient.

Examination costs at specialist departments were calculated for patients who had specific examinations. Examinations were counted and monetized using charges as determined by the recommendations of the Ministry of Health.

Total costs per patient resulted from the aggregation of inpatient-days’ costs multiplied by the length of stay, laboratory test costs, drug costs, medical material costs, admission costs, and costs of examinations at specialist departments. Example of calculating total costs for a patient is presented in the Additional file 1.

### Sources of data

Labor costs (wages and allowances) were quantified based on financial records of the hospital. Material (not charged to patients directly) and capital (both medical and non-medical equipment and building costs) were quantified based on the financial records, administration of materials used and capital inventories of the hospital.

Age, sex, department, length of stay, total numbers of each laboratory test, numbers of each drug and medical materials charged to patients directly were retrieved from patient-based databases of the hospital. Prices for specific services such as medication, materials/disposables and laboratory tests were available from the specific databases of the hospital. Furniture and land costs were not included in this study as no information was available.

The number of tests completed for each patient and its charges were obtained from the individual patient sheets. We selected all tests, including test for the diagnosis and/or treatment of hypertension and other condition. Total number of all tests in 6 months was multiplied by 2 and then divided by the total number of patients in 2011, equating 11 in the whole hospital. For the hypertensive in-patient group, the average number of tests per patient was 16.8, 16.3 and 17.1 in both groups, I10 and I10 + E75, respectively. Of course for costing, each individual test was priced separately.

### Statistical and sensitivity analysis

Means, medians and Inter Quartile Range (IQRs) of costs were measured as outcome in this study. Univariate sensitivity analyses were performed to explore the robustness of the analyses [18]. Notably, hypertensive patients may be admitted to hospital with comorbidities.

In this context, it is very important to rule out the costs resulting from diagnosing and treating these other diseases. Therefore, we conducted one sensitivity analysis with a scenario that excluded all costs resulting from diagnosing and treating comorbid diseases. We limited to specific costs for diagnosing and treating I10 and E75 diseases in this scenario. In addition, the discount rate for capital was varied from 1% to 5%. As data on furniture were not available, these costs were not included in the base case. Additionally, a scenario where an estimated 5% furniture cost was added to the total capital cost was explored [19]. Finally and in the absence of standardized national prices, we analyzed sensitivity t o laboratory tests by using laboratory test charges instead of laboratory test costs as used in the base case. Multivariate sensitivity analyses were also performed to explore the contribution of two or three parameters at once to the uncertainty in the total costs.

### Ethical issues

This study was approved by the ethical committee of the Thai Nguyen General hospital. Databases of this study were used under the permission of the Planning department of the Thai Nguyen General hospital.

## Results

The analyses were based on 230 patients who met the inclusion criteria, including 147 cases with essential (primary) hypertension (I10) and 83 cases with hypertension combined with sphingolipid metabolism and other lipid storage disorders (I10 + E75). Mean age was 64.3 (SD +/-14.7) and 53.5% was female. Characteristics of patients are indicated in Table 1. Median length of stay was 6 days with an IQR of 3-8 in the whole study population, and 5 days (IQR: 2-7) and 7 days (IQR: 5-9) in the in the I10 and I10 + E75 groups, respectively.

**Table 1:** **Characteristic of patients**

**Characteristic**	**n = 230**	**Percentage (%)**
Gender		
Female	123	53.5
Male	107	46.5
Patient group		
I10	147	64%
I10 + E75	83	36%
Age (mean ± SD)	64.3 (±14.7)	

Hypertensive patients were admitted in 3 departments of the hospital; i.e., Cardiovascular Internal Medicine, Geriatric Internal Medicine and Neurology. For these departments, base- case inpatient– day costs were US$4.99, US$5.05 and US$5.33, respectively. Notably, the most expensive per day costs were associated with patients admitted to Neurology department.

Costs of treatment for these hypertensive inpatients are presented in Table 2 as the result of the base case, which considers a 3% discount, uses laboratory test charges and includes no furniture costs. Median total direct costs per patient were US$65 (IQR: 37-95). Total costs per patient were higher in the combined hypertensive and lipid population (US$78) than in the sole hypertensive patients (US$53). However, the median costs per day were slightly higher in the sole hypertensive patients compared to the combined group with estimates of US$11.4 and US$10.9, respectively. In the base-case, inpatient-day costs, at 41% of the total cost, represented the highest cost component. Costs of drugs followed as second largest cost component with 34%, as presented in Table 2.

**Table 2:** **Direct costs (US$) per hypertensive inpatient by disease classification and cost category**

**Cost category**	**Median (and IQR) of costs**			**Mean (± SD) of costs**			**% of total costs of all patients**	**% of total costs of I10 group**	**% of total costs of I10 combine E75**
	**All patients (n = 230)**	**I10 group (n = 147)**	**I10 combined E75 group (n = 83)**	**All patients (n = 230)**	**I10 group (n = 147)**	**I10 combined E75 group (n = 83)**			
Inpatient days	29.94 (14.97 - 39.92)	24.95 (9.98 - 34.93)	34.93 (24.95 - 44.91)	29.05 (±16.45)	24.86 (±15.94)	36.48 (±14.69)	41.37	40.01	43.14
Drugs	11.67 (4.63 - 39.76)	8.26 (3.69 - 37.02)	16.13 (8.47 - 51.47)	23.85 (±25.4)	21.24 (±25.26)	28.45 (±25.12)	33.96	34.19	33.65
Tests	13.00 (6.89 - 19.24)	11.56 (5.62 - 15.76)	13.69 (7.81 - 25.76)	15.84 (±13.88)	14.49 (±13.46)	18.22 (±14.37)	22.55	23.33	21.54
Others	1.23 (0.84 - 1.68)	1.17 (0.82 - 1.56)	1.41 (0.85 - 1.83)	1.49 (±2.26)	1.53 (±2.79)	1.41 (±0.6)	2.12	2.47	1.67
Total costs	64.95 (37.18 - 95.32)	53.41 (29.71 - 82.82)	77.64 (56.61 –106.5)	70.23 (±42.89)	62.13 (±43.76)	84.56 (±37.46)	100	100	100

**Figure 1:**
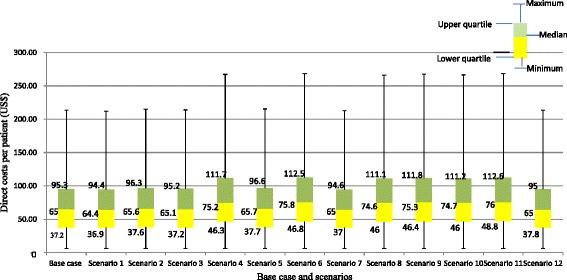
**Estimates of base case and scenario analyses for direct costs of hypertensive inpatients in Vietnam.** Notes: Scenario 1: 1% discounting for capital costs. Scenario 2: 5% discounting for capital costs. Scenario 3: Adding a 5% furniture cost to total capital costs. Scenario 4: Using laboratory test charges instead of cost prices. Scenario 5: 5% discounting for capital costs and adding a 5% furniture cost to total capital cost. Scenario 6: 5% discounting for capital costs and using laboratory test charges. Scenario 7: 1% discounting for capital costs and adding a 5% furniture cost to total capital costs. Scenario 8: 1% discounting for capital costs and using laboratory test charges. Scenario 9: Adding a 5% furniture cost to total capital costs and using laboratory test charges. Scenario 10: 1% discounting for capital cost, adding a 5% furniture cost to total capital costs and using laboratory test charges. Scenario 11: 5% discounting for capital cost, adding a 5% furniture cost to total capital costs and using laboratory test charges. Scenario 12: Separating out costs resulting from diagnosing and treating comorbid diseases.

Results of the sensitivity analyses, including 12 scenarios reflecting relevant alternative options, are presented in Figure 1. In all scenarios, inpatient-day costs appeared to be the most important cost driver in the total costs per patient. In the scenario where costs of comorbid diseases were excluded, the median total direct costs per patient were US$64.6 (IQR: 37 -95). Using laboratory testing charges instead of costs showed changes in the specific scenarios with increases between 15% and 17% (or US$75 to US$76 for total cost) compared to the base-case. Other scenarios, such as changing discount rates to 1% and 5%, adding 5% furniture to the capital costs produced minor impact on the total costs (from minus 0.8% to 1.1% changes in total costs).

## Discussion

Hypertension is a major and increasing public health problem in Vietnam, including 5.7 million patients unaware of their status, 2.1 million aware but untreated, 2.0 million treated but uncontrolled and 1.2 million treated and controlled [8]. Notably, the proportion of patients treated and controlled is modest, illustrating a potential for improvement. Vietnam spends huge amounts of money on these groups, especially when they need hospitalization for untreated and uncontrolled hypertension. The present study is the first to estimate the direct medical hospital costs of hypertensive inpatients treatment (including ICD-codes I10 or I10 combined with E75) in Vietnam. In this study, we both present both mean and median costs in the relevant table.

Our analysis estimates that the median total cost of inpatient treatment and care is US$65 per hypertensive patient per hospital stay. To put this in perspective, the total health expenditures in Vietnam per capita in 2010 was only US$83 [12]. As costs of inpatient treatment of hypertension are high, less costly options such as programs for earlier detection, treatment and subsequent control may avert these high hospitalization costs. For example, an earlier study demonstrated that cost of drug treatment at a community health station was US$9.4/patient/year for grade 1 hypertension and US$27/patient/year for grade 2 and 3 hypertension [11]. When comparing those figures to US$53 for hospitalization of uncontrolled sole hypertension in this study, the potential value of early drug treatment over hospitalization of uncontrolled diseases if evident. Hospitalization for uncontrolled hypertensive patients was two times and over five times the costs of drug treatment for grade 2 or 3 and grade 1 hypertension, respectively. The high cost for inpatient care for hypertension in Vietnam is consistent with a previous study in the Philippines, in which the lowest median hospitalization costs (US$57) were reported for essential or secondary hypertension among all hypertension-related hospitalization costs [5].

As expected, the costs of the sole hypertensive patients (US$53) were lower than the costs for the combined hypertensive and lipid patients (US$78). It is quite plausible that comorbid conditions can drive up costs, particularly due to increases in length of stay, compared to a single disease condition. In this study, median lengths of stay were 5 and 7 days for I10 and I10 combined with E75, respectively. These inpatients days were associated with high costs, representing 41% of the total cost. This finding differs from the study conducted in the Philippines, which reported medication costs (34% of the total) as the highest cost component [5]. However, this comparison between Vietnam and the Philippines may only have limited validity due to differences in perspectives and methods to estimate costs, differences in drug prices, and differences in overall investments in health care and health-care systems between both countries.

In this study, the number of inpatient days for sole-hypertensive patients was 5 days. These patients may have undetected and uncontrolled hypertension and now requires hospitalization to be diagnosed for primary or secondary hypertension. In addition, there is no ambulatory blood-pressure monitoring service available in the community. Patients must be admitted to a hospital for diagnosis. As an alternative to expensive hospital care, community level health services may serve as a cheaper option, but these centers need to be strengthened to enable blood-pressure monitoring for the broader population.

The present findings must be interpreted in the context of potential limitations. Firstly, the study was conducted in one hospital, which may not be representative hospitals across the country. However, it is a governmental and not-for-profit hospital that is potentially similar to other hospitals regarding governmental investments in hospital beds [17]. Thus, inpatient-day costs may be extrapolated to other hospitals, which have similar investments with the explicit notice that costs of each patient may be different as different care needs exist among patients. Secondly, we could not identify the grade of hypertension nor the exact type of sphingolipid metabolism or lipid storage disorder so the association between costs and seriousness of disease could not be made at such detailed level, or even at the level of exact blood pressure values. However, others have suggested that the level of systolic or diastolic blood pressure is not a valid predictor for cost outcomes [20]. Thirdly, we may have excluded some patients who were sole hypertensive or had combined lipid disorder but were not identified with I10 (combined) codes. Fourthly and conversely, there may be patients who were miscoded for I10 or E75, and thus incorrectly included in our analysis. We cannot estimate the size of this problem. However, it is reasonable to assume that it is small and likely random and would therefore not introduce specific biases in our study. Furthermore, we calculated tests’ costs based on the assumption the that total numbers for each test in quarters 3 and 4 would be equal to the total numbers in quarters 1 and 2, with lack of data for the whole year. Notably, some seasonality in testing may have some influence on our results. Finally, while available data did not allow for measurement of cost per day at each department for each disease as well as detailed admission costs and costs of examination at specialist department, we did perform specific analyses assuming price weights, reflecting the average of all patients in the department. However, this implies that the estimate may be artificially under- or overestimate costs.

## Conclusions

In comparison to annual medication treatment at a community health station for hypertension and total health expenditure per capita in Vietnam, the costs of hospitalization of hypertensive patients is high. The main driver of the costs is related to inpatient days rather than to treatment, laboratory or other cost categories. Our findings have important implications for health policies. Costs of treatment for hypertension and combined disorders of sphingolipid metabolism and other lipid storage disorders in this study could become the reference case for reimbursement when health insurance companies apply reimbursement by fee for diagnostic-related groups. The findings in this study, particularly the high cost of hospitalization for untreated and uncontrolled hypertension, justify increasing current expenditures by the Vietnamese health-care sector on effective interventions to control high blood pressure, which may produce savings to the health care budget by preventing expensive complications.

## Additional file

Additional file 1:
**Example of calculating total costs for a patient.**
