# Supplementary material for: Direct costs of hypertensive patients admitted to hospital in Vietnam– a bottom-up micro-costing analysis
Source: BMC Health Serv Res. 2014 Oct 28;14:514. doi: 10.1186/s12913-014-0514-4 (PMC4221683; doi:10.1186/s12913-014-0514-4)
Supplement: Additional file 1: — Example of calculating total costs for a patient. [file 12913_2014_514_MOESM1_ESM.docx]

Example of calculating total costs for a patient:

“Consider a male patient admitted to the Cardiovascular Internal Medicine Department for 4 days with ICD code I10+E75. He used ultrasound, an ECG-test and total laboratory test charges were US$8. The prescriptions included antihypertensive and lipid modifying agents with total drug charges at market prices (paid by the hospital) at US$7. Needles and gloves were used at US$1. Admission cost was equal to US$2 for every patient and inpatient-day costs in this department equaled US$5. He did not have any specific consultations and exams at any specialist department.

Total costs for this patient:

inpatient-days costs (5*4)+laboratory test costs (8) + drug costs (7)+costs of medical materials charged to patients directly (1)+ admission cost (2) + costs of examinations by specialists (0)

= US$38
